# Optical Fiber Vibration Signal Identification Method Based on Improved YOLOv4

**DOI:** 10.3390/s22239259

**Published:** 2022-11-28

**Authors:** Jiangwei Zhang, Jiaqing Mo, Xinrong Ma, Jincheng Huang, Fubao Song

**Affiliations:** Xinjiang Key Laboratory of Signal Detection and Processing, School of Information Science and Engineering, Xinjiang University, Urumqi 830017, China

**Keywords:** distributed optical fiber vibration sensor, object detection, YOLOv4, deep separable convolutional network, focal loss function

## Abstract

In the traditional peripheral-security-early-warning system, the endpoint detection and pattern recognition of the signals generated by the distributed optical fiber vibration sensors is completed step-by-step and in an orderly manner. The method by which these two processes may be placed end-to-end in a network model and processed simultaneously to improve work efficiency has increasingly become the focus of research. In this paper, the target detection algorithm combines the endpoint-detection and pattern-recognition processes of the vibration signal, which can not only quickly locate the start and end vibration positions of the signal but also accurately identify a certain type of signal. You Only Look Once v4 (YOLOv4) is one of the most advanced target detection algorithms, achieving the optimal balance of speed and accuracy. To reduce the complexity of the YOLOv4 model and solve the dataset’s unbalanced sample classification problem, we use a deep separable convolution (DSC) network and a focal loss function to improve the YOLOv4 model. In this paper, the five kinds of signals collected in real-time are visualized as two different datasets in oscillograph and time-frequency diagrams as detection objects. According to the experimental results, we obtained 98.50% and 93.48% mean Average Precision (mAP) and 84.8 and 69.9 frames per second (FPS), respectively, which are improved compared to YOLOv4. Comparing the improved algorithm with other optical fiber vibration signal recognition algorithms, the mAP and FPS values were improved, and the detection speed was about 20 times faster than that of other algorithms. The improved algorithm in this paper can quickly and accurately identify the vibration signal of external intrusion, reduce the false-alarm rate of the early-warning system, and improve the real-time detection rate of the system while ensuring high recognition accuracy.

## 1. Introduction

**Figure 1:**
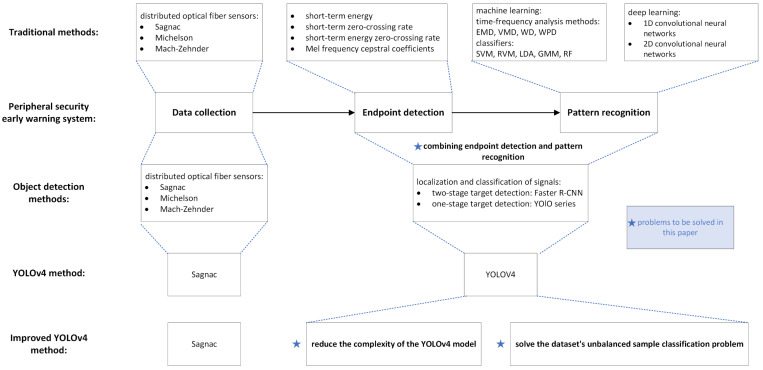
The problem solved in this paper and the method proposed.

The operation of the peripheral security early-warning system is roughly divided into three processes: firstly, when the external vibration occurs, the distributed optical fiber vibration sensor generates vibration signals, and the signal data we need is obtained through analog-to-digital conversion and digital signal processing; secondly, the received signal is processed by the endpoint-detection algorithm to remove unnecessary silent signals and noise; and finally, the signal is characterized by extraction and classification identification, which can accurately determine which kind of intrusion signal to achieve an early-warning effect. Figure 1 shows the realization process of the optical fiber vibration signal identification method which moved from traditional machine learning to one-dimensional and two-dimensional convolutional neural networks, then to object detection; moreover, the method in this paper is proposed according to the problem needing to be solved.

In the process of generating the signal, the external vibration will cause the local refractive index of the sensing fiber of the distributed fiber-optic vibration sensor to change, thereby changing the phase of the light wave propagating inside the fiber [1]. Therefore, interference or coherent detection techniques are usually used to acquire the signal. Commonly used distributed optical fiber sensors for perimeter security include Sagnac, Michelson, and Mach-Zehnder [2,3,4]. Compared with other types of sensors, Sagnac sensors have been widely used in fiber-optic vibration sensing systems due to their reciprocal structure and strong anti-interference ability [5]. Therefore, this paper adopts the Sagnac optical fiber sensor to collect the optical fiber vibration signal.

In the process of signal endpoint detection, the signal generated by external vibration will contain a large amount of redundant information, which will interfere with the judgment of valid information. The practical information is mainly concentrated in the vibration section of the signal. Therefore, it is necessary to perform endpoint detection on the signal and use the algorithm to locate the start and end points of the vibration to distinguish the vibration signal from the non-vibration signal. The endpoint-detection algorithms commonly used in vibration signals include short-term energy, short-term zero-crossing rate, short-term energy zero-crossing rate, and Mel-frequency cepstral coefficients [6,7,8]. Short-term energy distinguishes vibration signals from non-vibration signals according to different energy distributions. Yet, when the vibration signal is weak, the signal’s amplitude is small, and it is difficult to use the energy as a threshold to judge. The short-term zero-crossing rate is based on the number of times the signal passes through the zero value in a short period to evaluate different signals. Yet, when the background noise is intense, it is easily confused with the vibration signal, making it difficult to distinguish. The short-term energy zero-crossing rate calculates the values of high energy, low energy, and zero-crossing rate as the threshold. Its discriminating conditions are stricter than the other methods, and its detection effects of vibration signals with different frequencies are somewhat different, so it is not easy to achieve unity. Mel-frequency cepstral coefficients convert the spectrum into Mel-spectrum. Signals not significantly different on the spectrum may have large differences on the Mel-spectrum and are easy to distinguish. However, the method of Mel-frequency cepstral coefficients can only be applied to particular fields, such as speaker recognition and radar monitoring, and is not widely used in endpoint detection of optical fiber vibration signals. When using the above methods, conditions such as the amplitude of the signal’s vibration, the strength of the background noise, the change of the vibration frequency, and the scope of the applicable field must be considered. With the rapid development of computer vision, object detection is now being applied in more industrial fields [9]. The target detection algorithm can locate the position of different targets in a specific area. The target object only needs to consider its position, size, and shape in the entire region and does not need to study the target itself. Using the methods outlined in this paper, it is not necessary to extensively consider the characteristics of the signal itself, reducing many of the obstacles and improving the efficiency of endpoint detection.

In the process of signal pattern recognition, that is, signal feature extraction and recognition classification, most algorithms are based on machine learning and deep learning to identify which type of input signal belongs. Common time-frequency analysis methods include empirical mode decomposition (EMD), variational mode decomposition (VMD), wavelet transform (WT), and wavelet packet transform (WPT). Classifiers include the support vector machine (SVM), relevance vector machine (RVM), linear discriminant analysis (LDA), Gaussian mixture model (GMM), and random forest (RF) [10]. However, machine learning methods require manual feature extraction, which may result in the loss of information. For nonlinear regression tasks, the processing complexity is high, and the processing time is extended. For deep learning, the convolutional neural network (CNN) can realize automatic feature extraction and recognition classification of signals. Compared with machine learning, it simplifies the overall algorithm analysis and learning process and uses a deeper network structure to obtain more semantic information. The methods of using CNN are roughly divided into two types: one is to directly send the signal into a one-dimensional convolutional neural network (1D-CNN) after preprocessing [11], while the other is to increase the dimension of the signal and convert it into the form of a picture as the input of a two-dimensional convolutional neural network (2D-CNN) [12]. Since the CNN is mainly used in image classification tasks, in most cases, the 2D-CNN will get better classification results than the 1D-CNN. However, the simple 2D-CNN model can only classify a single target object in the picture, that is, for only one target in an image; yet, the simple model is no longer applicable when the picture contains multiple targets. The target detection algorithm can use a more complex network structure to deal with more difficult classification tasks. In this paper, we show that when multiple vibration positions generate different types of signals, the target detection algorithm can locate the vibration segments of the signal in real-time and identify the signal type, which speeds up the entire identification process.

In this paper, the YOLOv4 model and its improved model are applied to the target detection of optical fiber vibration signals. The signal’s endpoint-detection and pattern-recognition processes are combined into an end-to-end network model [13]. The target detection algorithms can be roughly divided into two categories. One is the two-stage target detection based on the candidate Region convolutional neural network (R-CNN) and its various derivative algorithms. They select multiple candidate regions through different algorithms and then input the target in each candidate region into CNN for feature extraction, classification, and bounding box regression [14]. The other type is the one-stage target detection based on the YOLO model and its improved model, using down-sampling components and splicing components or path aggregation networks to complete the feature extraction of original images of different dimensions, directly mapping from features of different sizes to target probabilities, boundaries box locations, and object classification probabilities [15,16]. YOLOv4 is one of the most advanced algorithms of YOLO, developed from continuous improvement in the original model. It can achieve the optimal detection speed and accuracy balance and can be embedded in actual machinery and equipment. Since YOLOv4 is only a network model designed for public datasets, its feature extraction network is more complex. The problem of unbalanced sample classification remains in the optical fiber vibration signal dataset developed in this paper. Therefore, based on the YOLOv4 model, the findings of this paper improve its backbone feature extraction network and loss function. The final experimental results show that the improved model not only makes the network computation smaller, that is, a lightweight model, but, additionaly, for the dataset of the signal time-frequency diagram, mAP and FPS are increased by 1.05% and 39.6, respectively. For the dataset of the signal oscillograph, these are increased by 2.3% and 41.9, respectively, both of which can quickly and accurately identify intrusion vibration events. The method proposed in this paper provides three significant contributions to the identification of optical fiber vibration signals:

1. The YOLOv4 target detection algorithm is used to complete the location and classification of vibration signals simultaneously, which means that it is no longer necessary to complete the feature extraction and recognition classification of signals by the pattern-recognition method after the traditional endpoint-detection algorithm. This can avoid not only the loss of signal information caused by the conventional endpoint-detection algorithm due to signal noise and other factors, but also improve the efficiency of signal recognition;
2. The deep separable convolution (DSC) network is used to replace the backbone feature extraction network in the YOLOv4 model, which makes the calculation amount of the improved model far less than that of YOLOv4. Additionally, it improves the recognition accuracy and detection rate of the signal;
3. The focal loss function is used to replace the confidence loss function in YOLOv4, and the problem of unbalanced sample distribution in the dataset is solved by changing the loss weight, which makes the recognition accuracy of signal types with more samples higher, and the recognition accuracy of signal types with fewer samples is also improved.

## 2. Distributed Optical Fiber Vibration Sensing System

**Figure 2:**
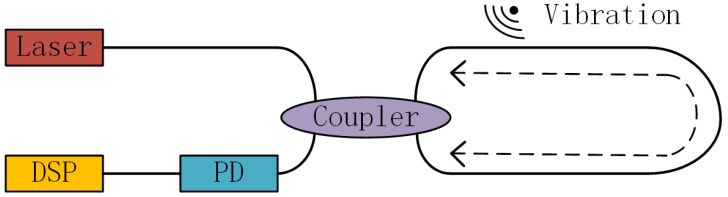
Working principle of Sagnac sensor.

The working principle of the Sagnac distributed optical fiber vibration sensor is shown in Figure 2. The light-source laser’s light is split into two beams with a constant phase difference through the coupler. Subsequently, the two beams of light enter the fiber ring to propagate clockwise and counterclockwise. When there is vibration in the outside world, the two beams of light pass through the vibration position at different times, resulting in an inconstant phase difference. Therefore, when the two beams of light end in the fiber ring and return to the coupler, the interference intensity changes, the optical signal is acquired by the photodetector (PD), and the final fiber-vibration-signal data is obtained through the digital-signal-processing process. In this paper, based on the field-test results, the sensing range of the distributed optical fiber vibration sensing system based on Sagnac can reach 10 km, and the spatial resolution is 30 m.

## 3. Dataset

### 3.1. Data Collection

**Figure 3:**
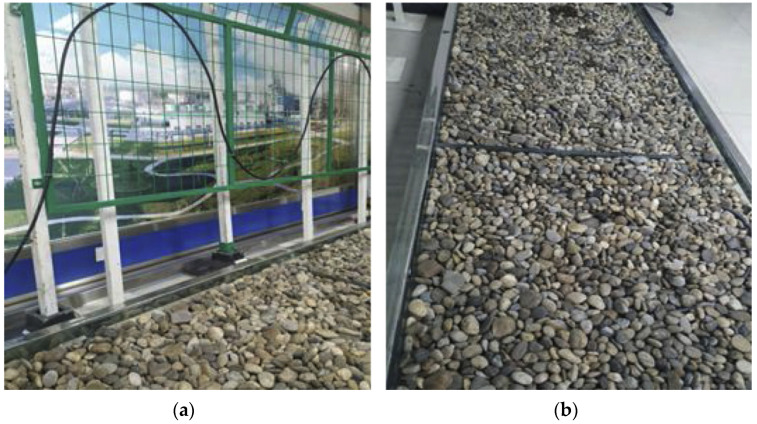
Laboratory testing environment; (**a**) hanging net; (**b**) and buried.

**Figure 4:**
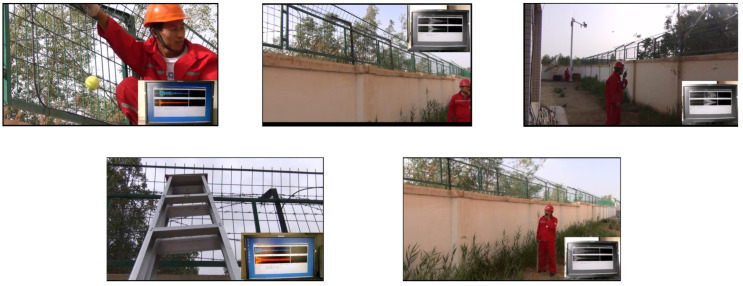
Field acquisition of partial vibration signals.

The data in this article come from Xinjiang Meite Intelligent Security Engineering Co., Ltd.’s (Urumqi, China) peripheral security early-warning system based on the Sagnac distributed optical fiber vibration sensor. This system combines hanging nets and buried structures to collect and detect optical fiber vibration signals. Figure 3a shows the hanging-net test environment, and Figure 3b shows the buried test environment. As shown in Figure 4, we used a variety of different operation methods as intrusion events. After the vibration passes through the early-warning system, the collected vibration signal is finally displayed using the display. The collected dataset contains five types of signals: flapping, knocking, stone- throwing, running, and walking. In this study, we collected 47 records. Each record is about 20 min, takes about 30 s as a sample, and guarantees that at least one complete signal exists in each sample. To improve the generalization ability and robustness of the model, we performed data-enhancement processing by adding noise, flipping, and time-shifting to the signal without changing the characteristics of the signal itself. The total number of signal samples collected in the end was 7544, which were used for model training, validation, and testing, where the ratio of the total number of samples in the training set plus the validation set to the total number of samples in the test set was 9:1, and the number of samples in the training set and the validation set was also distributed by 9:1. It is worth noting that the data augmentation processing in this paper only occurred in the training set, and the other datasets remain unchanged. Table 1 shows the number of each signal type.

### 3.2. Oscillograph and Time-Frequency Diagram

**Figure 5:**
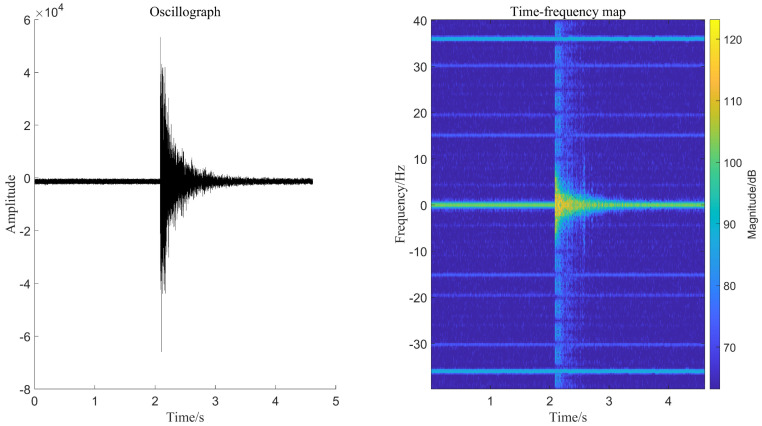
Oscillograph and time-frequency diagram.

Most of the collected signal datasets are in the form of binary or decimal text, and the changes in amplitude, frequency, and peak value of the signal cannot be directly observed. The oscillograph is an image representing any particle curve, and the principle of the oscilloscope is derived from this. The density of the oscillograph in a certain period can demonstrate the frequency level, and the distance of the particle away from the original position can be seen as the change of the amplitude value. The shape of each oscillograph is inconsistent, meaning the envelope is different. These differences can be used to identify different types of signals. The time-frequency diagram is an image generated by the time-frequency analysis method, which can observe the joint information of the time and frequency domains; that is, it can reflect the signal frequency change with time. The difference in color in the time-frequency diagram indicates the inconsistency of the energy density of the frequency. This paper converts the signal into a time-frequency diagram through a short-time Fourier transform. Different signals are judged using the characteristics of interlaced colors in the time-frequency diagram [17]. Therefore, as shown in Figure 5, we converted the collected signal datasets into two forms: oscillograph and time-frequency diagram and set the size of each graph to 416*416 as the target detection algorithm’s input to observe which dataset has better recognition performance.

### 3.3. Signal Labeling

**Figure 6:**
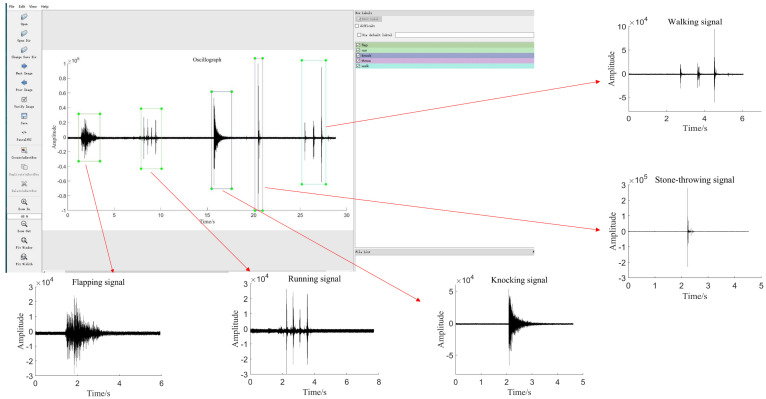
Annotation of the oscillograph dataset.

When using the YOLO series of target detection algorithms, the requirement for the dataset is to include the original image and the type and position coordinate information of the marked target in the picture, as shown in Figure 6. In this paper, the Labellmg annotation tool was used to mark the signal’s oscillograph and time-frequency diagram, and each image’s marked information was saved in an XML file. Due to the different vibration laws of the five types of signals, the marking methods were also different. The whole vibration trajectory can be easily marked for three kinds of signals with short vibration time, such as flapping, knocking, and stone-throwing. However, for walking and running signals, they are composed of multiple vibration segments and last for a long time. The research on a single vibration signal is of little significance, and the law of the entire signal cannot be seen, so it has to label a whole segment of the signal. Labeling the time-frequency diagram is similar to the oscillograph, and it should ensure the signal’s integrity as much as possible.

## 4. Materials and Methods

### 4.1. YOLOv4

#### 4.1.1. Innovation of YOLOv4

YOLOv4 is the continuous improvement and perfection of the YOLO series of target detection algorithms. Due to its high detection accuracy and fast detection speed for target objects, it is widely used in various industries. YOLOv4 has made some small innovations to the details of YOLOv3 [18], but the entire prediction-network framework has not changed. The main improvements are: (1) the backbone feature extraction network was changed from Darknet53 to CSPDarknet53, and the activation function was changed from LeakyReLU to Mish; (2) the enhanced feature extraction network was changed from the feature pyramid network (FPN) to spatial pyramid pooling (SPP), and the path aggregation network (PANet); (3) Mosaic is used for data augmentation; and (4) the loss of bounding box regression was changed from intersection over union (IOU) to complete intersection over union (CIOU).

#### 4.1.2. The Network Structure and Prediction Principles of Yolov4

**Figure 7:**
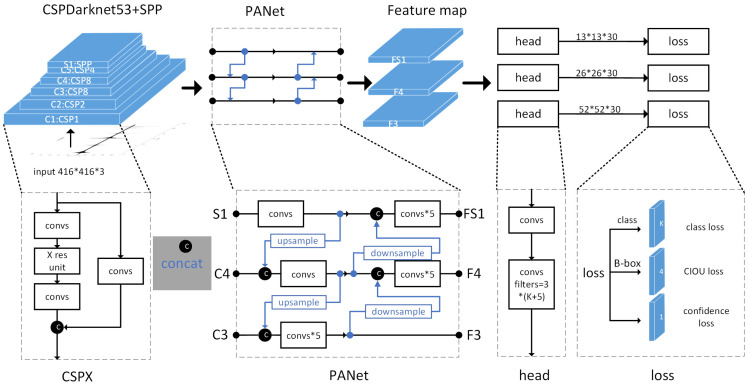
Flowchart of the network structure of YOLOv4.

The network structure of YOLOv4 is shown in Figure 7. This network comprises three parts: backbone feature extraction network, enhanced feature extraction network, and feature prediction. The backbone feature extraction network uses the residual module in CSPDarknet53 to continuously halve the size of the input image and reduce the pixels, and its purpose is to extract more feature information in the picture. The enhanced feature extraction network consists of SPP and PANet. The function of SPP is to increase the receptive field and isolate the most significant context features. PANet uses the process of upsampling and downsampling to repeatedly extract features and enhance shallow features through feature fusion; three enhanced features are obtained in the feature prediction part, and the loss function with the actual data can be calculated, as well as the predicted bounding box, target category, and its confidence value.

#### 4.1.3. Loss Function

The loss function of YOLOYv4 consists of classes loss, confidence loss, and bounding box loss. The formula of the loss function is as follows:

(1) $$Loss\left(bounding\;box\right)=\sum_{i=0}^{s^{2}}\sum_{j=0}^{B}I_{ij}^{obj}\left(2-w_{i}^{j}\times h_{i}^{j}\right)\left(1-CIOU\right)$$

(2) $$\begin{array}{ll} Loss\left(confidence\;\right)= & -\sum_{i=0}^{s^{2}}\sum_{j=0}^{B}I_{ij}^{obj}\left[C_{i}^{j}log\left(\hat{C_{i}^{j}}\right)+\left(1-C_{i}^{j}\right)log\left(1-\hat{C_{i}^{j}}\right)\right]\\ & -\sum_{i=0}^{s^{2}}\sum_{j=0}^{B}I_{ij}^{noobj}\left[C_{i}^{j}log\left(\hat{C_{i}^{j}}\right)+\left(1-C_{i}^{j}\right)log\left(1-\hat{C_{i}^{j}}\right)\right] \end{array}$$

(3) $$\begin{array}{ll} Loss\left(classes\right)= & -\sum_{i=0}^{s^{2}}\sum_{j=0}^{B}I_{ij}^{obj}\sum_{c\;\in \;classes}[P_{j}^{i}\left(c\right)log(\hat{P_{j}^{i}}\left(c\right))\\ & +\left(1-P_{j}^{i}\left(c\right)\right)log\left(1-\hat{P_{j}^{i}}\left(c\right)\right)] \end{array}$$

(4) $$Loss=Loss\left(bounding\;box\right)+Loss\left(confidence\;\right)+Loss\left(classes\right)$$

where ***S*** represents the grid size of an output feature, ***B*** represents the number of anchor boxes, and each output feature has three corresponding anchor boxes, ***B*** = 3. $I_{ij}^{obj}$ indicates whether the anchor box in any grid contains the target to be detected; if so, it is equal to 1; if not, it is equal to 0. ***w*** and ***h*** represent the width and height of the ground-truth box, $2-w_{i}^{j}\times h_{i}^{j}$ represents the penalty term, and the smaller the value is, the closer the predicted box is to the ground-truth box. ***CIOU*** is calculated by the overlapping area of the predicted box and the ground-truth box, the center point distance, and the aspect ratio. $C_{i}^{j}$ indicates the confidence of the actual label, either 1 or 0.

#### 4.1.4. Prediction of Bounding Boxes and Confidence Values

**Figure 8:**
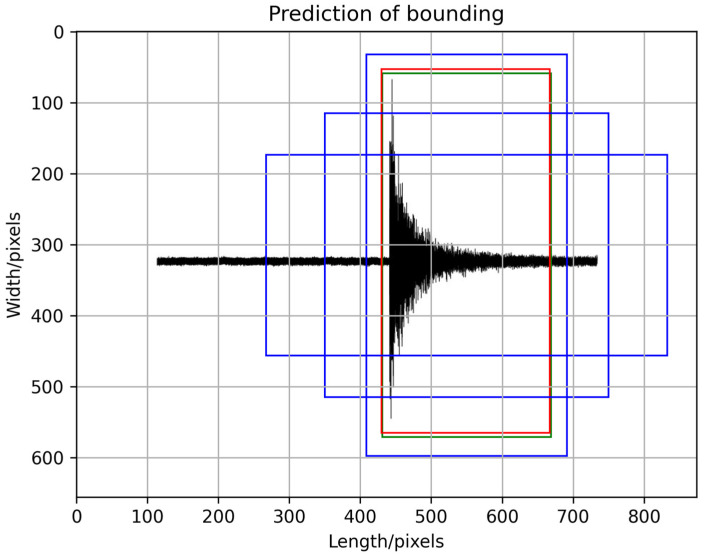
Prediction of bounding boxes. The blue box represents the set anchor box, the green box represents the ground-truth box, and the red box represents the predicted bounding box.

In the prediction results of YOLOv4, the bounding box is used to locate the target, and the classification confidence value is used to represent the predicted probability of the target category. The prediction of the bounding box is related to the set anchor box size and the three feature information outputs from the network. The size of the anchor box is selected based on experience, also called a priori box. The prediction information output by the network adjusts the position of the prior box to get the predicted bounding box. Figure 8 shows the process of generating prediction boxes with test signals. To better highlight the color of the box, the signal waveform is adjusted to black. The three blue boxes in the figure are anchor boxes generated according to the target center point. Only one of these three can generate a prediction box, so it is necessary to calculate the maximum IOU value of the anchor box and the ground-truth box, that is, the anchor box that overlaps the most with the ground-truth box. The green box represents the ground-truth box, and the red box represents the predicted bounding box. To calculate the coordinate information of the final predicted bounding box, we set the width and height of the anchor box mapped to the feature map as $p_{w},p_{h}$, respectively, and the coordinates of the predicted box as $b_{x},b_{y},b_{w},b_{h}$. The coordinates of the ground-truth box were set as $g_{x},g_{y},g_{w},g_{h}$, and the feature information outputs from the network were set as $t_{x},t_{y},t_{w},t_{h}$, so we have:

(5) $$t_{x}=g_{x}-c_{x}$$

(6) $$t_{y}=g_{y}-c_{y}$$

(7) $$t_{w}=log\left(\frac{g_{w}}{p_{w}}\right)$$

(8) $$t_{h}=log\left(\frac{g_{h}}{p_{h}}\right)$$

where $c_{x},c_{y}$ is the position relative to the upper left corner of the grid, that is, the size of each grid, $t_{x},t_{y}$ represents the predicted coordinate offset, and $t_{w},t_{h}$ represents the scale scaling. Once these parameters are obtained, the coordinates of the predicted bounding box can be found:

(9) $$t_{x}=g_{x}-c_{x}$$

(10) $$b_{x}=\sigma \left(t_{x}\right)+c_{x}$$

(11) $$b_{w}=p_{w}e^{t_{w}}$$

(12) $$b_{h}=p_{h}e^{t_{h}}$$

where $t_{x},t_{y}$ uses the sigmoid function to make the offset in the range of 0~1. After adding $c_{x},c_{y}$, the center coordinate $b_{x},b_{y}$ of the predicted bounding box can be obtained, and $t_{w},t_{h}$ can get the height and width of the predicted bounding box by acting on the height and width of the anchor box. In general, the information learned by the network is used to adjust the position parameters of the anchor box to make it closer to the ground-truth box. After the bounding box is predicted, the confidence value can be used to indicate whether the current box contains a target and to determine the accuracy of the predicted bounding box coordinates. The formula is as follows:

(13) $$Pr\left(Object\right)*IOU_{pred}^{truth}$$

when there is a target in the bounding box, $pr\left(Object\right)$ is equal to 1. Otherwise, it is equal to 0, and the confidence value at this time is the IOU value of the prediction box and the ground-truth box. The probability of the target category is the conditional probability of the class multiplied by the confidence value, so the classification confidence value is calculated as follows:

(14) $$Pr\left(Class_{i}|Object\right)*Pr\left(Object\right)*IOU_{pred}^{truth}=Pr\left(Class_{i}\right)*IOU_{pred}^{truth}$$

At this point, all bounding boxes and classification confidence values for each target prediction will have been obtained. However, the prediction result will have many repeated bounding boxes in the same target. A non-maximum suppression (NMS) algorithm is proposed to ensure the optimal result. First, set the confidence threshold and IOU threshold, then use the confidence threshold to eliminate bounding boxes with lower scores, arrange the confidence of the remaining bounding boxes from high to low, and finally calculate the IOU values of the box with the highest confidence and the other boxes. The larger the IOU value, the greater the degree of overlap between the two boxes, and the predicted bounding box that is greater than the IOU threshold will be removed.

**Figure 9:**
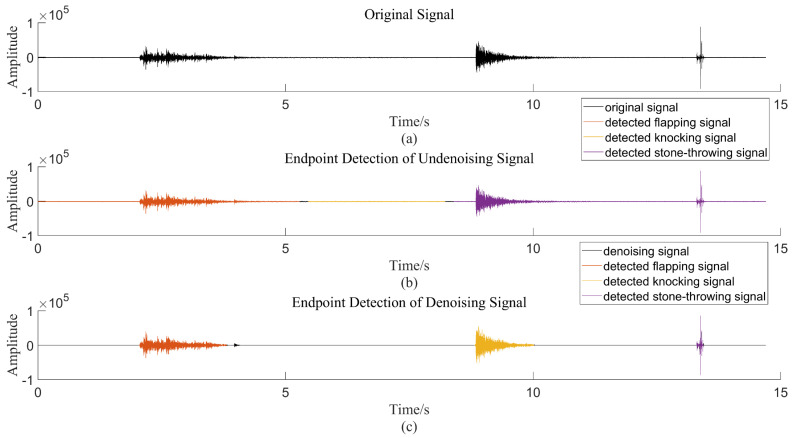
Endpoint detection method based on short-term energy combined with spectral spread. (**a**) Original Signal; (**b**) Endpoint Detection of Undenoising Signal; (**c**) Endpoint Detection of Denoising Signal.

The idea of the predicted target location in the YOLOv4 algorithm is similar to the endpoint-detection algorithm of the traditional optical fiber vibration signal. The purpose is to locate the vibration segment of the signal. This paper proposes an endpoint detection method based on short-term energy and spectral spread. First, the characteristic sequences of short-term energy and spectral spread are extracted from the vibration signal, and then the two characteristic sequences are judged by the estimated threshold. Finally, according to the judgment result, the vibration segment, the silent segment, and the noise segment of the vibration signal are distinguished. As shown in Figure 9, we mark the detected signals using different colors. This method incorrectly classifies the knocking and stone-throwing signals as the same type of signal to the original signal without filtering noise. It can be seen that the results of the traditional endpoint detection method are not only greatly affected by noise but, additionally, a pattern-recognition algorithm is needed to identify the type of the signal after the vibration signal is extracted, and the experimental process is more complicated. For the YOLOv4 target detection algorithm, when the noise in the signal is small, the prediction result of the bounding box will not be affected without denoising the signal, which reduces the experimental steps. In the final prediction result, the type of the signal can also be discriminated, which realizes the unification of the endpoint detection and pattern recognition of the signal and improves the recognition efficiency of the whole system.

#### 4.1.5. Evaluation Indicators

The performance of an algorithm needs to be measured by evaluation indicators. The leading evaluation indicators for the YOLOv4 algorithm are Precision, Recall, F1-score, mAP, and FPS. Their calculation formulas are as follows:

(15) $$\mathrm{Precision}=\frac{TP}{TP+FP}$$

(16) $$\mathrm{Recall}=\frac{TP}{TP+FN}$$

(17) $$F1\text{-}\mathrm{score}=\frac{2\times \mathrm{Precision}\times \mathrm{Recall}}{\mathrm{Precision}+\mathrm{Recall}}$$

(18) $$\mathrm{mAP}=\frac{1}{N}\sum_{i=0}^{N}\int_{0}^{1}{\left(\mathrm{Precision}\times \mathrm{Recall}\right)}_{i}$$

(19) $$\mathrm{FPS}=\frac{FrameCount}{ElapsedTime}$$

where ***TP*** indicates the predicted class is true and the actual class is also true; ***FP*** indicates the predicted class is true and the actual class is false; and ***FN*** means the predicted class is false, and the actual class is true. Therefore, **Precision** indicates how many samples are correctly predicted for the predicted samples, which is used to measure the degree of false detection, and **Recall** shows how many samples in the actual samples are correctly predicted, which is used to measure the degree of missed detection. For the evaluation index of an algorithm, we hope that the results for Precision and Recall are at a high level, so the **F1-score** is used as the weighted average of these two indicators to evaluate the algorithm’s quality comprehensively. In the above formula, $\int_{0}^{1}{\left(\mathrm{Precision}\times \mathrm{Recall}\right)}_{i}$ represents the Average Precision (***AP***) of the ***i*** category, and **mAP** represents the average accuracy of all categories ***N***, which is used to measure the quality of the learned model for all classes. **FPS** indicates how many frames (pictures) can be detected per second, which is used to measure the detection speed of the target-detection network. The larger the **FPS**, the faster the processing speed. It is worth noting that the values of Precision and Recall will change due to the different IOU between the prediction box and the ground-truth box, so a uniform threshold value (IOU is generally set as 0.5) will be set before calculation. For example, mAP50 is the value calculated under the condition that IOU equals 0.5. The larger the IOU value, the more accurate the prediction box is required to predict, and the smaller the mAP value.

### 4.2. Improved YOLOv4

The advantages of the YOLOv4 target detection algorithm’s fast detection speed and high detection accuracy are all trained and tested on public datasets. When used on self-created datasets, the results are often worse than expected, and there is an imbalanced sample classification in the dataset. Therefore, based on such considerations, this paper improves the YOLOv4 model, and the improvement is mainly in two aspects: (1) the improvement in the backbone feature extraction network; and (2) the improvement in the loss function.

#### 4.2.1. Improvement in Backbone Feature Extraction Network

**Figure 10:**
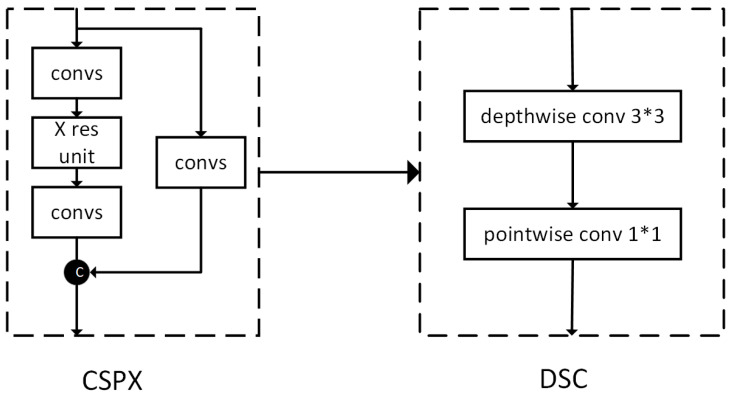
DSC module replaces CSP module.

The backbone feature extraction network CSPDarknet53 of YOLOv4 is mainly composed of stacking CSP modules [19]. The network structure of CSP consists of two parts: the backbone network output which features a convolution block and a residual block, and the other network which passes through a convolution block, similar to the residual edge of the residual network. The two extracted features are subsequently fused. As shown in Figure 10, in the CSP module, besides the residual block, the blocks are all convolution blocks, and too many convolution blocks will significantly increase the computational load of the model, resulting in a long training time and low detection rate. Compared with the CSP module, the overall structure of the depthwise separable convolution (DSC) module is very simple, as there is no mosaic residual edge structure, reducing the difficulty of writing the code; furthermore, there are no redundant convolutional layers in the DSC module, which reduces the network model, meaning that network models stacked by DSC modules are smaller, easier to train, and thus less computationally expensive, requiring fewer parameters when extracting the same number of features. Therefore, to make the backbone feature network more lightweight, this study replaced the CSP module with a DSC module [20].

The convolution process of ***DSC*** is divided into depthwise convolution (***DC***) and pointwise convolution (***PC***). ***DC*** uses a convolution kernel to perform a convolution on each channel of the input image, that is, to obtain feature maps with the same number of input channels but different sizes. At this time, each channel is relatively independent, and the features are relatively scattered. To change the number of output channels and enhance the connection between each channel, the ***PC*** can fuse the features output by different channels so that the features are enhanced, and the number of output channels is controlled by changing the number of convolution kernels. ***DSC*** can achieve the effect of ordinary convolution. The difference between them lies in the calculation formula. The ordinary convolution is:

(20) $$C_{CONV}=I_{w}\times I_{l}\times F_{w}\times F_{l}\times M\times N$$

***DSC*** is:

(21) $$C_{DSC}=C_{DC}+C_{PC}\;=I_{w}\times I_{l}\times F_{w}\times F_{l}\times M+I_{DC}{}_{w}\times I_{DC}{}_{l}\times F_{PC}{}_{w}\times F_{PC}{}_{l}\times M\times N$$

The ratio of the two parameters is:

(22) $$\frac{C_{DSC}}{C_{CONV}}=\frac{1}{N}+\frac{I_{DC}{}_{w}\times I_{DC}{}_{l}\times F_{PC}{}_{w}\times F_{PC}{}_{l}}{I_{w}\times I_{l}\times F_{w}\times F_{l}}$$

where ***I*** represents the input image, ***F*** represents the convolution kernel, ***M*** represents the number of input channels (image dimension), and ***N*** represents the number of output channels (the number of convolution kernels). In the ratio of Equation (22), the feature map after ***DC*** is smaller than the original image size $I_{w}\times I_{l}$, and in general, $F_{PC_{W}}=F_{PC_{l}}=1$. Therefore, the calculation amount of ***DSC*** is much smaller than that of ordinary convolution, which means that when the amount of calculation is the same, ***DSC*** can use a deeper number of layers to construct the network architecture, thereby improving the accuracy of the model predictions.

**Figure 11:**
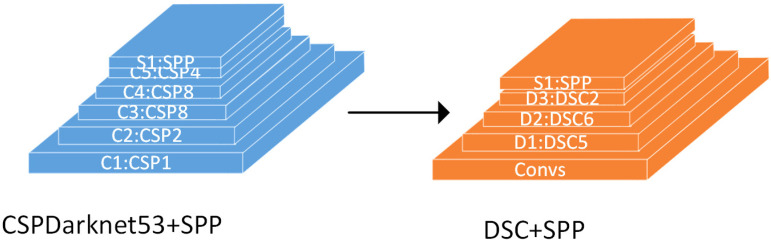
Improved backbone feature extraction network.

The backbone extraction feature network used in this paper, which utilized the stacking of ***DSC*** modules, is shown in Figure 11. To ensure that the size of the extracted enhanced-feature layer and the number of channels remain the same as those of CSPDarknet53, one layer of convolution blocks and five layers of ***DSC*** shoud be gone through before extracting the first feature layer, six layers of ***DSC*** should be gone through before extracting the second feature layer, and finally two layers of ***DSC*** and SSP layers should be gone through before extracting the third feature layer. This is a process of continuously compressing the input image and increasing the number of channels to extract more features from the original image. It is worth noting that the model performance is better when a batch normalization layer and an activation function layer are not added to each ***DSC*** module.

#### 4.2.2. Improvement in Loss Function

Because the YOLOv4 detector does not have a sub-network used specifically to generate candidate boxes, it is unable to reduce the number of candidate boxes to a relatively small order of magnitude; therefore, the vast majority of candidate boxes are background classes, greatly distracting the energy on non-background classes and leading to an extremely unbalanced proportion of positive and negative samples. Furthermore, the gradient is dominated by simple negative samples. We refer to the background class as negative samples. Although the loss caused by a single negative sample is very small, their overall contribution to the loss is still dominant due to the extremely large number of negative samples. This leads to a convergence that is less than a good result. As the number of negative samples is large and the YOLOv4 detector cannot reduce the number of negative samples, it is natural to reduce the weight of negative samples and make positive samples occupy more weight so that the training will focus on the samples that are more meaningful than the others, which is the role of focal loss.

**Figure 12:**
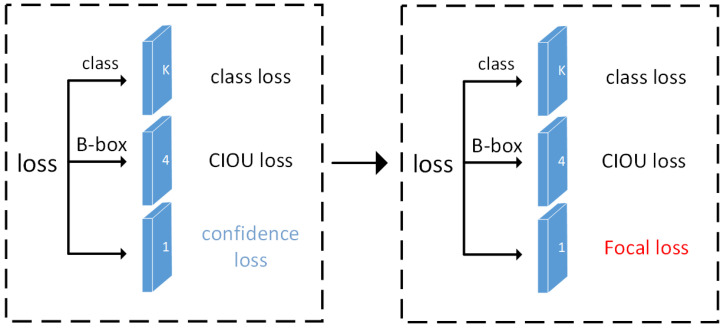
Focal loss function replaces the confidence loss function in YOLOv4.

Therefore, to solve the problem of the accuracy of model prediction not beideal due to the imbalanced sample classification in the dataset, we changed the confidence loss in the YOLOv4 loss function to focal loss [21], as shown in Figure 12. The focal loss function was used to change the loss weight of positive samples and negative samples, and the loss weight of hard-to-distinguish samples and easy-to-distinguish samples. The positive samples and negative samples were judged according to whether there were prior boxes in the target’s ground-truth box. The hard-to-distinguish samples and easy-to-distinguish samples were judged according to the predicted probability size of a particular class of samples. The focal loss function is as follows:

(23) $$Focal\;Loss\left(p,\alpha ,\gamma \right)=-log\left(p\right)\times \alpha \times {\left(1-p\right)}^{\gamma }$$

where p represents the prediction probability of the sample, α represents the weight coefficient of the balanced positive samples and negative samples, and γ represents the weight coefficient of the balanced hard-to-distinguish samples and easy-to-distinguish samples. When the sample is positive, p indicates that the sample is easy-to-distinguish (the larger the p value, the easier the classification); that is, the ground-truth box is distributed with priori boxes, and a large number of priori boxes will be generated in the target detection, only a tiny part of which will be distributed in the vicinity of the ground-truth box. This leads to a severe imbalance in the proportion of positive and negative samples, so the value of α is controlled to adjust the weight of positive samples, so that the proportion of positive samples becomes larger, and the value of γ is controlled to attenuate the loss of easy-to-distinguish samples. Similarly, when the sample is negative, and p represents hard-to-distinguish samples, the proportion of negative samples is reduced by controlling the value of α, and the loss of hard-to-distinguish samples is attenuated by controlling the value of γ. There are two points worth noting here. Firstly, the loss attenuation degree of the same γ value differs between the hard-to-distinguish samples and the easy-to-distinguish samples. Compared with the easy-to-distinguish samples, the attenuation degrees of the hard-to-distinguish samples are smaller, which makes the loss proportion of the hard-to-distinguish samples heavier. Hence, the model pays more attention to the hard-to-distinguish samples. Secondly, because γ dominates, as it increases, α decreases. Therefore, the focal loss function not only solves the problems of having few learning features, poor training efficiency, and model degradation caused by too many prior boxes, but also makes the dataset pay more attention to the few and hard-to-distinguish class samples in the training process. The focal loss function was applied in this study. After many experiments, when α = 0.25, γ = 2, the final model prediction accuracy was the best.

## 5. Results and Discussion

### 5.1. Environment Configuration and Training Parameter Settings

For the improved YOLOv4 model, there was no difference between the environment configuration and training parameter settings of YOLOv4. The entire preliminary data processing was conducted on the Matlab and Pycharm platforms, and the experimental results were trained and run on Google Colaboratory. Due to the limitation of GPU resources on Google Colaboratory, we divided the training process into a freezing phase and an unfreeze phase to meet the training needs. In the freezing phase, we set the epoch to 50 times and the batch size to 16. In the unfreeze phase, we set the epoch to 300 times, that is, the training times of the entire model, and the batch size to 8. The initial learning rate was set to 0.01, and the learning rate was reduced using cosine annealing decay [22], but the minimum learning rate was limited to 0.01 times the initial learning rate. Other parameter settings: the optimizer used SGD, momentum was equal to 0.937, and weight decay was equal to 0.0005.

### 5.2. Training Results and Analysis

**Figure 13:**
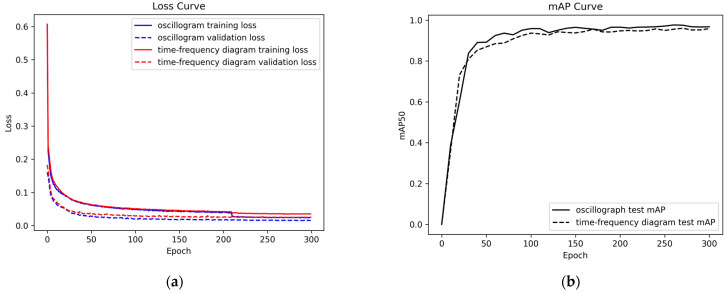
The curve change of the dataset; (**a**) loss curves for the training and validation sets; and (**b**) mAP curves for the test sets.

The training loss and validation loss curves for the two datasets are shown in Figure 13a. Regardless of the kind of dataset, the overall loss curve first dropped sharply, then slowly decreased, and finally stabilized. For the oscillograph, the training loss and validation loss stabilized around 0.02 and 0.01, respectively, while the training loss and validation loss for the time-frequency diagram stabilized around 0.03 and 0.02, respectively. Figure 13b shows the mAP curve measured by the test set for the model saved during the training process. It can be seen that, similarly to the training loss and validation loss performance, the two test sets also achieved the expected effect on the model evaluation, obtaining the optimal mAP values of 96.20% and 98.50%. The gradual stabilization of the loss curve and the mAP curve indicate that the training model tends to converge, and the experimental results of the two datasets achieve the expected goals. In general, the oscillograph shows less loss and higher mAP, indicating that the model is albe to easily learn the feature information in the oscillograph.

The test sets of the two datasets were used to test the performance of the training model. To obtain better prediction results, the confidence threshold was set to 0.5, and the IOU threshold was set to 0.5. As shown in Table 2, for the oscillograph, the improved YOLOv4 model increased mAP, Precision, and FPS by 2.30%, 3.47%, and 41.9, respectively, and for the time-frequency diagram, it also increased these values by 1.05%, 4.38%, and 39.6, respectively. This shows that, regardless of the form of image input used, the improved model improved accuracy and real-time detection speed, and even the FPS was improved by almost two times when compared to the original. While ensuring higher accuracy, it also accelerates the processing efficiency of the entire model. However, for both datasets, the performance of the improved model was better on the oscillograph, therefore, only the oscillograph dataset is discussed in the following section.

### 5.3. Pretraining

**Figure 14:**
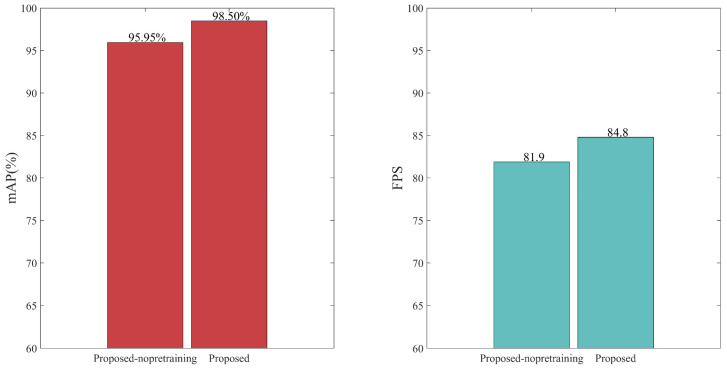
The impact of pre-training on the model.

Whether or not pretraining is performed before training impacts the experimental results. Pretraining is based on the idea of transfer learning, and the obtained weights can be used to train on different datasets because their characteristics are interlinked with datasets using the same feature extraction network model. Additionally, the use of pretrained weights makes the weights obtained by the network model less random, the feature extraction effect more noticeable, and the network training results better than those with no pretraining. Therefore, we used the improved YOLOv4 model to train on the VOC dataset 07 + 12, observed the change of the loss value of the training, and selected the weight of an optimal model for the training of the optical fiber vibration dataset. Figure 14 compares the experimental results of the improved model with and without pretraining weights. The use of pretraining weights increased the mAP value by 2.55% and increased the FPS value by about 3. Therefore, for the whole experiment, pretraining was necessary.

### 5.4. Comparison of Backbone Feature Extraction Networks

**Figure 15:**
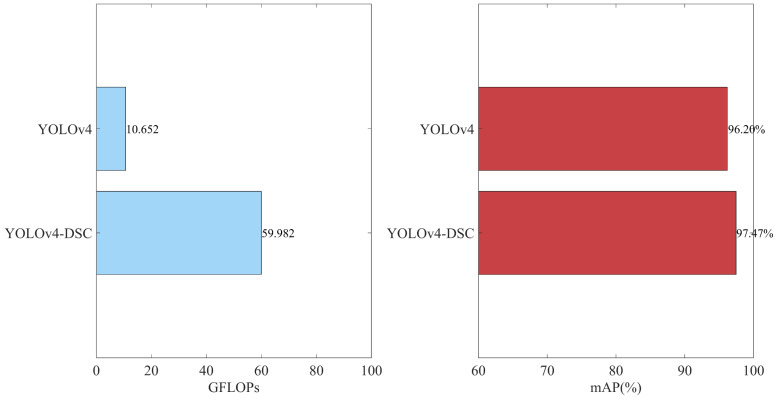
Comparison of YOLOv4 and YOLOv4-DSC.

The backbone feature extraction network model of YOLOv4 is especially suitable for training on large public datasets with a wide variety of samples. With regard to the datasets in this article, YOLOv4’s model is relatively complex and requires a large amount of computation, making it easy to waste unnecessary computing resources. Therefore, we replaced the feature extraction network of YOLOv4 with the DSC stacked network and used giga floating-point operations per second (GFLOPs) to represent the computational load of the model, which can be used to measure the complexity of the model [23]. As shown in Figure 15, the calculation amount of the improved model is one-sixth of that of YOLOv4, and the mAP was increased by 1.27%, indicating that the DSC network not only improves the accuracy of the model but also saves many network layers, making the network more lightweight.

### 5.5. Comparison of Loss Functions

**Figure 16:**
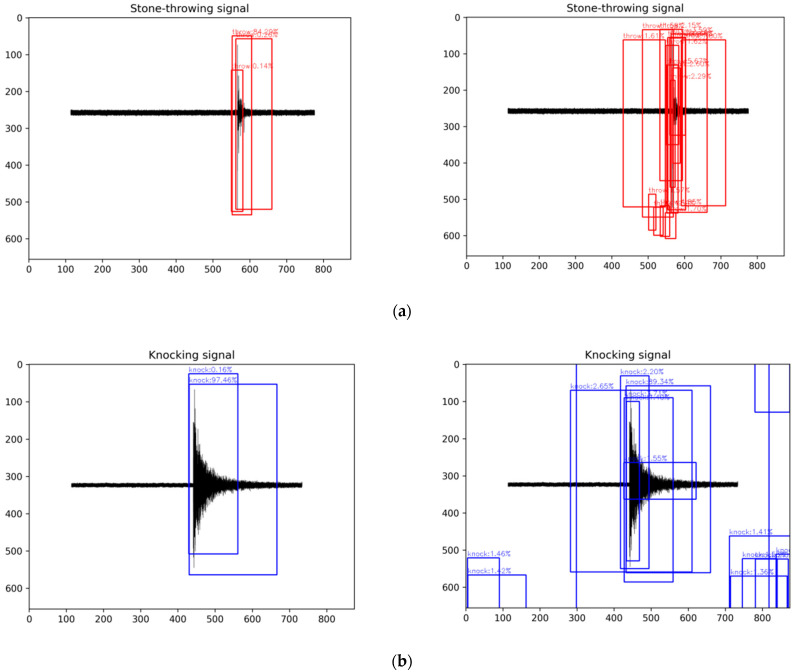
The prediction results of the focal loss function for two types of signals with large differences in sample distribution; (**a**) stone-throwing signal, the left side of the figure is the result predicted by the focal loss function, and the right side is the result predicted by the YOLOv4 confidence loss function; (**b**) the knocking signal and the left and right sides of the figure are the same as (**a**).

By replacing the confidence loss function in YOLOv4 with the focal loss function, the test effects of the two on the knocking signal (large number, easy-to-distinguish) and stone-throwing signal (small number, hard-to-distinguish) were compared. To meet the mAP values obtained from different thresholds, the bounding boxes of all categories and the classification confidence values were calculated in the prediction stage. As shown in Figure 16, for the stone-throwing signal, the loss function of YOLOv4 caused many useless and wrong bounding boxes to be generated, and compared with the knocking signal, the predicted bounding box positions were denser and confidence values were much closer, making it more difficult to distinguish. This result was mostly caused by too few anchor boxes containing the target and too few samples of the target, and the focal loss function can solve this problem by changing the weight. It can be seen from the experimental results that the use of the focal loss function not only improves the confidence value of the prediction of a small number of samples but also has a better effect on the prediction of a large number of samples.

### 5.6. Performance Comparison of the Overall Algorithm

**Figure 17:**
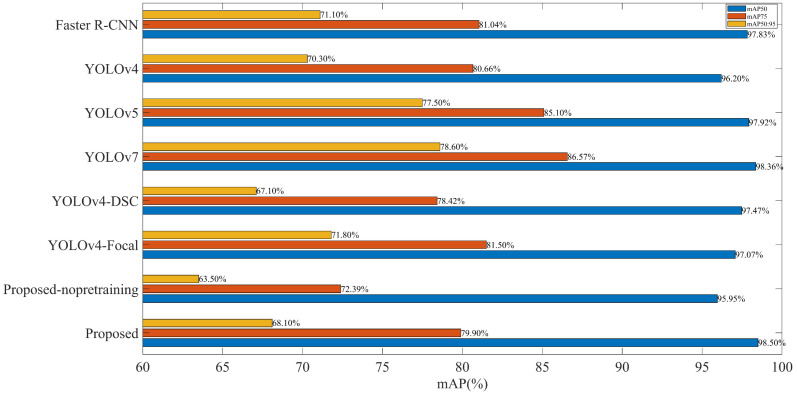
Comparison of mAP values of the different algorithms.

This study tried different improvements to the YOLOv4 network model. Table 3 shows the performance evaluation indicators of different algorithms. It can be seen that, compared with Faster R-CNN [24], YOLOv4 has a slight decrease in mAP value. Still, its FPS value is six times faster than that of R-CNN, and its GFLOPs is one-sixth of that of R-CNN, which shows that YOLOv4 can use a lighter network structure and faster detection speed to obtain almost the same accuracy. The improvements of YOLOv5 over YOLOv4 are mainly focused on the acquisition method of anchor boxes, data enhancement, activation function, NMS, loss function, and model optimizer. In contrast, the backbone feature extraction network, feature enhancement network, and prediction network of the whole model are consistent with that of YOLOv4. In terms of the experimental results, the values of each evaluation index of YOLOv5 were comprehensively improved compared with that of YOLOv4. Yolov5 is a detector with a small network structure, high accuracy, and fast inference speed. However, YOLOv5 is only the implementer of the YOLOv4 algorithm and does not make drastic changes to the network structure, so compared with the method proposed in this paper, its improvement space is not large. YOLOv7 is the most cutting-edge technology in the YOLO series, most of which is inherited from YOLOv5. YOLOv7 applies the latest advancements developed in recent years: efficient aggregation network, reparameterized convolution, auxiliary head detection, model scaling, etc. The experimental results show that YOLOv7 is 0.44% and 0.37% higher than YOLOv5 in mAP and Precision, respectively. However, in terms of inference speed, it is much slower than YOLOv4, YOLOv5, and the improved method in this paper, which is not consistent with the official performance of inference speed. This shows that in practical applications, the effect of the YOLOv7 algorithm is worth discussing. Compared with the two methods of only improving the feature extraction network of YOLOv4 and only improving the loss function of YOLOv4, the method proposed in this paper enhances the mAP value, Precision value, and FPS value to 98.50%, 99.73%, and 84.8, respectively. This shows that simultaneously applying the two improved methods to YOLOv4 can also improve the model’s performance. Using the advantages of the two methods, the model prediction is more accurate, the detection speed is faster, and the computing resources are less preoccupied. This further shows that, compared with YOLOv4, the improved model enhances the feature extraction ability, enables the network to extract deeper semantic information, and reduces the inference time of the model. Therefore, the detection effect on the given dataset is better, and the number of detected false prediction boxes is less, meaning the whole detector pays more attention to the target, thus performing the localization and classification tasks more accurately. Figure 17 illustrates the mAP values of each algorithm for IOU equal to 0.5, 0.75, and 0.5:0.95. It can be seen that, when the prediction box is required to predict more accurately, the focal loss function demonstrates its advantages in solving the problem of few samples and the difficulty of distinguishing samples in the dataset, making the position of the prediction box more accurate and the value of classification confidence higher. It is worth noting that, although the DSC module can reduce the computational complexity of the model, its model performance may not necessarily be improved under different conditions. In general, under the evaluation of various indicators, the performance of the method proposed in this paper is optimal overall compared with other methods.

### 5.7. Visual Comparison of Prediction Results

**Figure 18:**
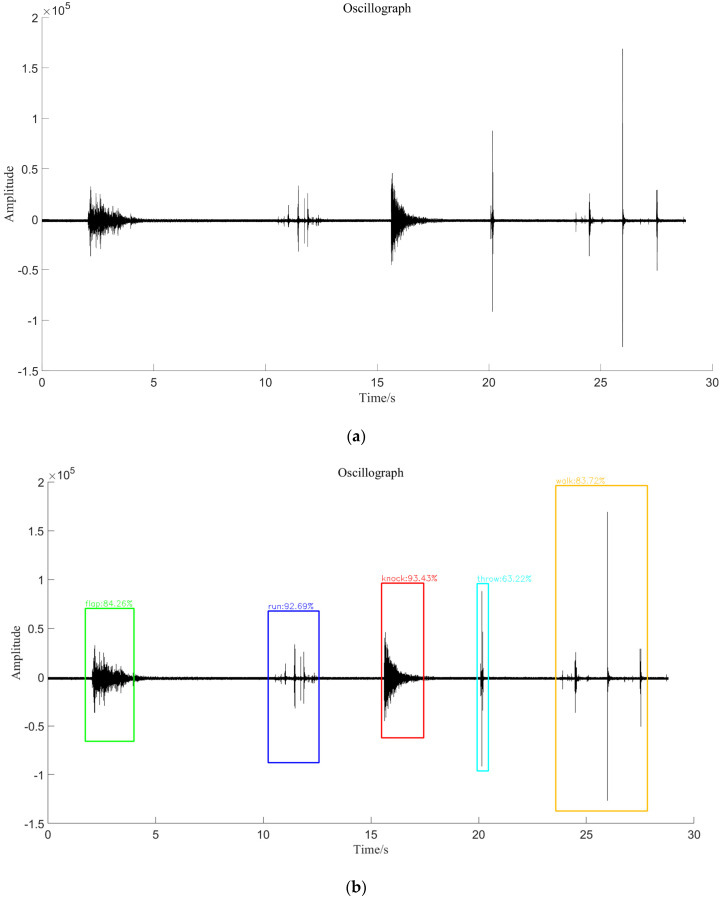

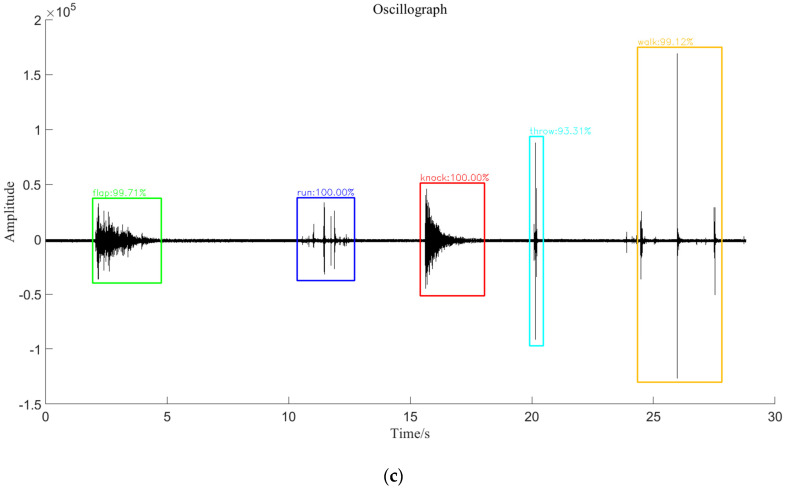
Comparison of the prediction results of different algorithms for each type of signal; (**a**) the original waveform; (**b**) the prediction result of YOLOv4; and (**c**) the prediction result of the method proposed in this paper.

In Figure 18, the visualization of the prediction results of each class of signals in the same signal segment is shown for both the YOLOv4 algorithm and its improved algorithm. We use different color boxes to represent different categories of signals, and it can be seen that each algorithm is able to effectively locate the vibration segment of the signal and identify to which vibration signal it belongs. However, in terms of the accuracy of positioning and the accuracy of signal category recognition, the proposed method is more refined. It reduces the segment length of the silent signal on the premise of ensuring the integrity of the vibration signal so that the detector pays more attention to the vibration segment of the signal.

### 5.8. Comparison with Other Identification Methods

Table 4 compares the experimental results between the four optical fiber vibration signal identification methods and the method proposed in this paper. In these four methods, the datasets are processed by the maximum value normalization before the subsequent feature extraction and identification classification work. The first method changes the matrix’s size based on the fact that the original dataset is a two-dimensional matrix and directly processes it using a suitable 1D-CNN. In the second method, after the step of changing the size of the matrix in the first method, it is transformed into a grayscale image using dimension enhancement and input into a 2D-CNN. The third method is an improvement in the first method. It uses wavelet packet transform (WPT) to decompose and reconstruct the signal matrix, filter the high-frequency and low-frequency parts of the signal, and fuse the generated wavelet packet coefficients into a two-dimensional matrix. The 1D-CNN is the same as the first method. The difference between the last method and the third method is that, after generating a two-dimensional matrix containing wavelet coefficients, the last method is converted into a grayscale image. Finally, the 2D-CNN in the second method is improved. Among these four methods, the model using WPT combined with 2D-CNN has the best performance. Despite this, these methods have a common disadvantage in that the speed of processing data samples is slow. The proposed method not only improves the recognition accuracy but also improves the detection speed by about 20 times compared with other methods, indicating that the model achieves a balance between recognition speed and accuracy. It is worth noting that other methods will carry out the processing of endpoint detection before training the signal dataset, which depends on the quality of the endpoint detection method and may cause the loss of signal information, resulting in a low recognition accuracy rate. The endpoint detection and pattern recognition of the signal by the method proposed in this paper are accomplished by predicting the position of the bounding box of the vibration segment of the signal and obtaining its classification confidence. These two processes are carried out simultaneously and form an end-to-end network model, which can improve the efficiency of the whole recognition process, and the obtained experimental results illustrate this point.

## 6. Conclusions

In this paper, five kinds of optical fiber vibration signals collected by the Sagnac distributed optical fiber vibration sensor are converted into two data sets: oscillograph and time-frequency diagram. The YOLOv4 target detection algorithm can locate different signals and identify their categories at the same time. This realizes the unification of traditional endpoint-detection and pattern-recognition algorithms and integrates two different algorithms into the same end-to-end network. The backbone feature extraction network and confidence loss function in YOLOv4 is improved by using the respective advantages of the DSC network and the focal loss function. The experimental results show that both the YOLOv4 model and its improved model can better identify the feature information in the oscillograph compared with the time-frequency diagram. For the improved model, the mAP corresponding to the system recognition accuracy, the Precision corresponding to the system false detection rate, the FPS corresponding to the system detection speed, and the GFLOPs corresponding to the system calculation amount have reached a satisfactory balance. Its detection performance is better than other experimental models. Compared with other optical fiber vibration signal recognition algorithms, it is found that the real-time detection speed of the algorithm proposed in this paper is much faster than other algorithms, which verifies the advantage of the YOLO-model detection speed. The visualization of the prediction results shows that the proposed method can quickly locate and accurately identify five kinds of external vibration signals in the surrounding security early-warning system, making it possible to embed it in mobile monitoring equipment.

There were many challenges throughout the experiment. When dealing with the signal dataset, the initial intention was to label the data text directly. However, we did not find a suitable method and chose to convert the data into pictures for subsequent recognition work. In terms of choosing a detector, the results from using YOLOv1, YOLOv2, and YOLOv3 are poor. Although some improvements have been made to these models, the results are always unsatisfactory. Therefore, this paper chooses YOLOv4, solves the problems in the experimental results of YOLOv4, and finally proposes an improved network model.

Future work should focus on mining more intrusion vibration events to improve the model’s performance and to identify similar events adaptively. By constantly testing the accuracy and detection rate of the model in the actual scene and continually improving and optimizing the results, the peripheral security early-warning system is more likely to identify abnormal intrusion events with insignificant vibration amplitude and short vibration time.

## Figures and Tables

**Table 1:** Number of various types of signals.

Signal Type	Knocking	Flapping	Running	Walking	Stone-Throwing
**Number of signals of each type**	7560	5516	3340	4604	2052

**Table 2:** Test results of different datasets (confidence threshold is 0.5 and IOU threshold is 0.5).

Datasets	Methods	mAP50	Precision	Recall	F1-Score	FPS	GFLOPs
Time-frequency diagram	YOLOv4	92.43%	90.14%	**89.82%**	**90.00%**	30.3	59.982
	Proposed	**93.48%**	**94.52%**	85.62%	89.85%	**69.9**	**10.652**
Oscillograph	YOLOv4	96.20%	96.26%	**95.20%**	**95.73%**	42.9	59.982
	Proposed	**98.50%**	**99.73%**	89.70%	95.45%	**84.8**	**10.652**

**Table 3:** Experimental results of different algorithms.

Faster R-CNN	YOLOv4	YOLOv5	YOLOv7	DSC	Focal Loss	No Pre- Training	mAP50	Precision	Recall	F1-Score	FPS	GFLOPs
**√**							97.83%	86.74%	**98.59%**	92.29%	7.8	370.210
	**√**						96.20%	96.26%	95.20%	95.73%	42.9	59.982
		**√**					97.92%	97.90%	97.10%	**97.50%**	64.5	17.156
			**√**				98.36%	98.63%	92.31%	95.37%	27.4	106.472
	**√**			**√**			97.47%	98.19%	94.92%	96.53%	64.6	10.652
	**√**				**√**		97.07%	96.82%	94.18%	95.48%	44.4	59.982
	**√**			**√**	**√**	**√**	95.95%	96.49%	81.92%	88.61%	81.9	10.652
	**√**			**√**	**√**		**98.50%**	**99.73%**	89.70%	95.45%	**84.8**	**10.652**

**Table 4:** Method comparison of different optical fiber vibration signals.

Methods	mAP	FPS
1D-CNN	83.74%	2.4
2D-CNN	76.93%	3.4
WPT-1D-CNN	91.92%	3.4
WPT-2D-CNN	96.66%	3.6
Proposed	**98.50%**	**84.8**

## Data Availability

The data presented in this study are available on request from the corresponding author.
